# Emotional recognition while watching emotional videos: Based on electroencephalography signal analysis and machine learning models

**DOI:** 10.1002/ibra.70002

**Published:** 2025-09-19

**Authors:** Afshin S. Asl, Sahar Karimpour

**Affiliations:** ^1^ Department of Biomedical Engineering, Faculty of Electrical and Computer Engineering University of Tabriz Tabriz Iran; ^2^ Department of Biology, Faculty of Natural Sciences University of Tabriz Tabriz Iran

**Keywords:** classification model, electroencephalography, emotion, emotional recognition, machine learning

## Abstract

Depending on the impact of emotions on a person's performance and emotional disorders that can be the main cause of many mental illnesses, as well as the desire of technology to design machines that are able to change their performance according to a person's emotional states, the study of electroencephalography (EEG) signals to analyze the different dimensions of human emotions has become increasingly significant. Based on machine learning models, this study was designed to identify the five emotions of relaxation, happiness, motivation, sadness and fear using EEG signal analysis. EEG data were collected from 23 male master's students at Tabriz University, aged 24–31, as they watched five videos designed to elicit different emotional responses. After preprocessing to remove noise and artifacts, we extracted statistical and frequency‐domain features from the raw signal. The features were labeled and selected using statistical tests. In the final step, five different emotions were classified using decision tree, linear discriminant analysis (LDA), Naive Bayes, support vector machine (SVM), K‐nearest neighbor (KNN), ensemble, logistic regression and neural network. It has been verified that ensemble and decision tree models had the highest accuracy with 95.38% and 91.77%.

## Graphical abstract

Overview of the EEG‐based emotion classification framework. The process starts with EEG signal recording while subjects watch five emotional videos. The raw EEG data undergoes preprocessing, including noise and artifact removal, followed by segmentation, feature extraction, and selection. The labeled data is then classified using machine learning models to detect five emotional states: relaxation, happiness, motivation, sadness, and fear.

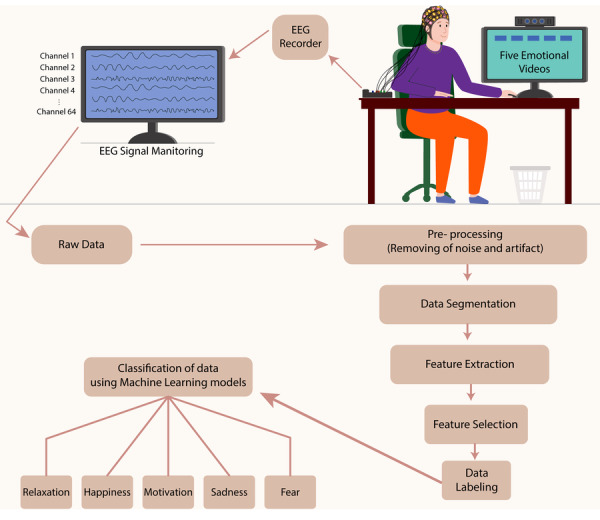

## 1 INTRODUCTION

Given the significant advancements in artificial intelligence, biophysics, physiology, and other emerging sciences, we can expect the emergence of intelligent technologies that can be adapted based on human emotions and thoughts. Considering recent quantum physics theories on the influence of energy, thoughts, and emotions on the external world, studying and understanding human brain waves and uncovering their connection to emotions has become a crucial necessity in today's world. Accurately classifying human emotions remains a challenging yet crucial task for applications in mental health and emotion‐aware technology.

Electroencephalography (EEG) is a technique used to measure the brain's electrical activity. Currently, EEG signals are utilized to diagnose and assess treatment outcomes for various neurological and brain‐related conditions, including Parkinson's disease, epilepsy, encephalitis, schizophrenia, and depression.^1^, ^2^ Beyond clinical diagnostics, EEG has also proven valuable in capturing the brain's response to emotional stimuli, offering a noninvasive means to analyze and quantify emotional states. Emotions play a crucial role in shaping human behavior and actions throughout life. With advancements in signal processing and neural networks, there is growing interest in developing systems and machines that can be adjusted based on human emotions. Emotion recognition is a key area where science and technology intersect, particularly in brain‐computer interface (BCI) applications. A good performance in BCI systems often relies on an effective emotion recognition system.^3^ Based on EEG, one study,^4^ using the DEAP database and the adaptive multilayer generalized learning vector quantization (AMGLVQ) algorithm, successfully classified four distinct emotional states with an accuracy of 62.98%.

Human stress is one example of the wide range of emotions experienced by individuals. A study published in 2019^5^ explored stress classification by recording EEG signals from 27 participants (14 males and 13 females) while they listened to music. The study used an entropy algorithm to categorize the stress levels. In another study, effective acoustic parameters for voice analysis were identified, showing a correlation with emotional changes.^6^ Additionally, a paper using correlation‐based subset selection successfully classified four emotional states with an accuracy of 82%.^7^ The Kernel Density Estimation algorithm is another method used for classifying EEG signals. In one study,^8^ this algorithm was employed to categorize the four primary emotional states.

Handling high‐dimensional data can be challenging, so feature extraction and dimensionality reduction techniques in signal processing and neural networks are crucial. For instance, a study^9^ used the synchrosqueezing transform for feature extraction from EEG signals to predict emotional states. Several other studies have applied the valence‐arousal‐dominance (VAD) model for emotion labeling. These studies utilized VAD dimensions to identify different emotions by combining fractal dimension features with statistical and higher‐order crossing (HOC) features.^10^, ^11^, ^12^

Although various methods, such as electromyogram signals, magnetic resonance imaging (MRI), and skin temperature, are used for recognizing emotional states, EEG signals are preferred due to their convenience and reliability. With the growth of body sensor networks,^13^, ^14^ the development of intelligent sensor systems,^15^, ^16^ and advancements in hospital systems,^17^ EEG signals have become increasingly popular for emotional state recognition. This is largely because EEG signals allow for easy modification and adjustment of algorithms, making them more versatile than other methods.

Recent research on emotion recognition has also employed methods like multivariate empirical mode decomposition (MEMD).^18^, ^19^, ^20^, ^21^, ^22^ Furthermore, emotions can be expressed in different forms, such as visual (images), audio‐visual (video clips), or audio (songs), and the method chosen for classification often depends on the specific goal.^23^ Additionally, research by Davidson and Fox suggests that frontal brain activity is linked to both positive and negative emotions.^24^, ^25^ Emotion classification has also utilized probabilistic classifiers and the “perceptron convergence” algorithm.^26^

Recent advancements in EEG‐based emotion recognition have demonstrated the effectiveness of various computational approaches. For instance, different feature extraction methods, including time‐domain, frequency‐domain, and nonlinear features, have been analyzed to enhance emotion recognition accuracy.^27^ Additionally, deep learning techniques such as deep belief networks, convolutional neural networks (CNNs), and recurrent neural networks have shown promising results in learning high‐level feature representations from EEG data.^28^ More recent studies have introduced novel modeling techniques to further improve EEG‐based emotion recognition by capturing spatial and temporal dependencies. However, many methods overlook the integration of prior knowledge for spatial dependencies and the modeling of cross‐temporal dependencies between consecutive time slices of different brain regions. To address these issues, Variational Spatial and Gaussian Temporal (VSGT) graph models have been proposed, effectively combining variational Bayesian and Gaussian graph transforms to dynamically model spatial and temporal dependencies, achieving superior performance on multiple EEG datasets.^29^ Furthermore, a model that integrates the efficient channel attention (ECA‐Net) module into a customized convolutional neural network (CNN) and gated recurrent unit (GRU), and utilizes four‐dimensional data encompassing temporal, spatial, and frequency information, has effectively addressed the challenges of capturing spatial dependencies among brain regions and temporal relationships across time slices, significantly improving classification performance in EEG‐based emotion recognition.^30^ Redwan et al. propose a hybrid CNN‐BiLSTM model for EEG‐based emotion recognition, leveraging Power Spectral Density (PSD) features to capture both spatial and temporal patterns in EEG signals. The CNN component extracts spatial features, while the BiLSTM enhances temporal context understanding, achieving a high classification accuracy of 97.5% for positive, negative, and neutral emotions.^31^ These advancements underscore the potential of integrating advanced signal processing techniques and deep learning models to improve the accuracy and reliability of EEG‐based emotion recognition systems. This study, based on machine learning, was designed to generate robust models to identify the five emotions using EEG signal analysis.

## 2 MATERIALS AND METHODS

### 2.1 Data set

Twenty‐three healthy postgraduate students aged 24–31 from Tabriz University volunteered for EEG signal recording test. The EEG signal was recorded using a 64‐channel device and the sampling rate of 250 Hz. The EEG signal is usually expressed in different frequency bands, which are: delta (0.5–4 Hz), theta (4–8 Hz), alpha (8–13 Hz), beta (13–30 Hz), and gamma (>30 Hz).^32^ Since most of the brain activity is in theta and alpha frequency bands while watching the video,^33^ and to avoid noises higher than 50 Hz, the EEG signal was recorded with low frequency and high frequency of 0.3 Hz and 50 Hz. More information about the experiment protocol is provided in Supporting Information S1: Table 1. This study was approved by the Ethics Committee of Tabriz University (test ethics code: IR.TABRIZU.REC.1398.030), and all participants provided written informed consent.

#### 2.1.1 Experimental procedure

**Figure 1:**
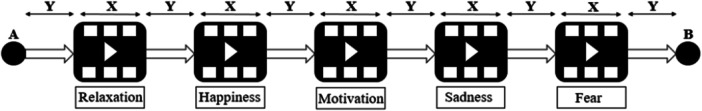
EEG signal recording setup. A represents starting point, while B represents finishing point. X denotes duration of video clips (5 min), and Y denotes duration of break periods (1 min).

This study aimed to identify five distinct emotions—relaxation, happiness, motivation, sadness, and fear—using EEG signal analysis. The experimental procedure was as follows: (1) Five videos, each designed to evoke a specific emotional state, were selected, with each video lasting 5 min. The descriptions and links of the videos are provided in Table 1. (2) A 1‐min break was introduced between each pair of consecutive videos to allow participants to recover emotionally. (3) A black screen was displayed for 1 min before the start and after the end of the videos to stabilize the baseline brain activity. (4) The total EEG recording duration was 31 min. A schematic representation of the EEG recording setup is presented in Figure 1.

**Table 1:** Descriptions of the videos used in experiment.

Emotional videos mode	Link of videos	Videos playback sequence number
Relaxation	https://youtu.be/vlrPhfrdges	1
Happiness	https://youtu.be/2v3mLxd2FfA	2
Motivation	https://youtu.be/uF55H3TD5sE	3
Sadness	https://youtu.be/5-CX1sAtTrc	4
Fear	https://youtu.be/dumK-do5ezc	5

To ensure that the emotional videos effectively elicited the intended affective responses, we incorporated a subjective assessment procedure following the EEG recording. After the completion of the 31‐min EEG session, each participant was asked to complete a brief questionnaire. This questionnaire evaluated the emotional impact of each of the five videos, based on the target emotional categories: relaxation, happiness, motivation, sadness, and fear.

Participants rated the intensity of each emotion experienced after watching each video using a 5‐point Likert scale, ranging from 1 (“not at all”) to 5 (“very strongly”). These self‐reported ratings allowed us to assess whether the emotional content of the videos was perceived as intended. The collected responses were used to verify the consistency of emotional elicitation across participants. This additional step helped validate the emotional categorization of the stimuli and ensured that the observed EEG patterns were aligned with the participants' subjective emotional experiences.

#### 2.1.2 EEG signal recording system

**Figure 2:**
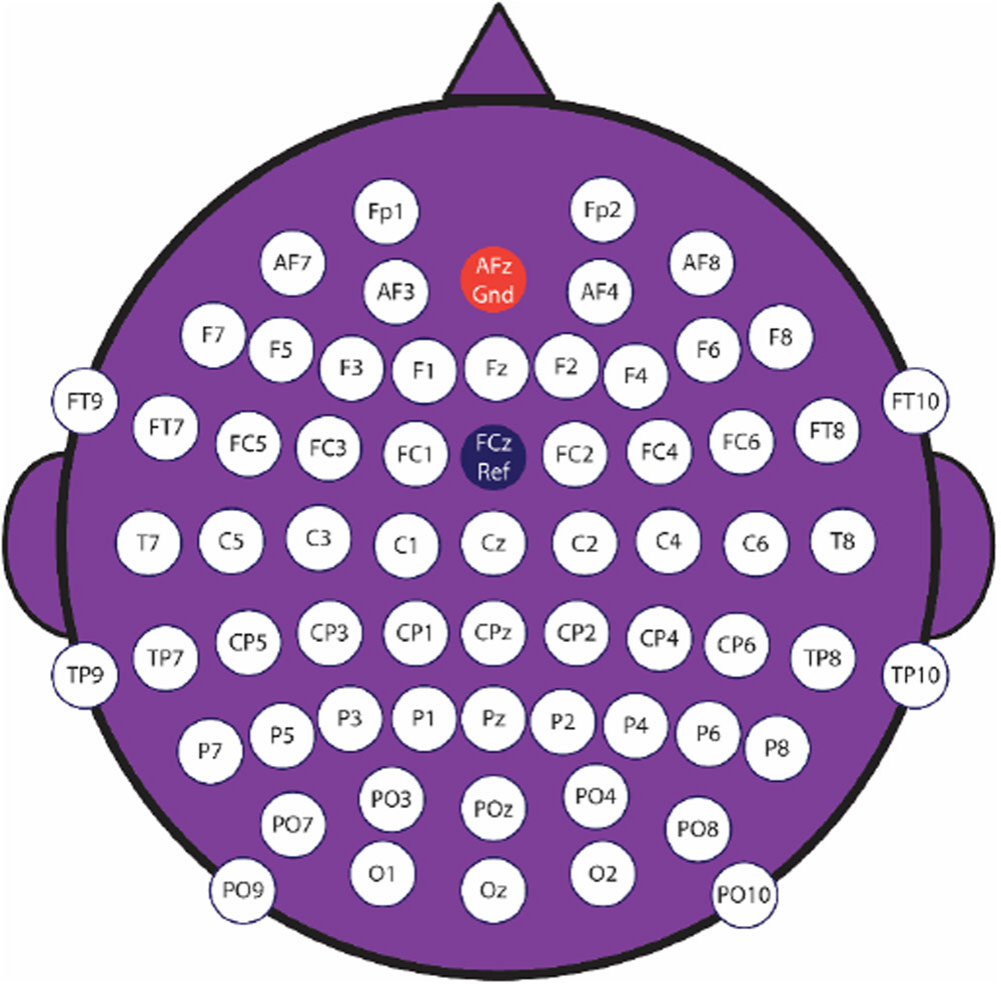
Locations of the channels. [Color figure can be viewed at wileyonlinelibrary.com]

Considering the timing diagram, the EEG signal was recorded when subjects were watching the videos. We used the international 10–20 system. In Figure 2, the channels location is given. Additionally, the number and labels of the channels used are listed in Supplementary Table 2.

### 2.2 Preprocessing of EEG data

#### 2.2.1 Filtering process

Filtering is a crucial preprocessing step in the analysis of EEG signals. Its primary objective is to reduce or eliminate frequencies that are irrelevant or considered noise. In regions like Europe, Asia, Africa, and Oceania, power line interference is present at 50 Hz. For this reason, a band‐pass filter is applied with a frequency range of 0.5–45 Hz, allowing us to capture frequency bands such as delta (0.5–4 Hz), theta (4–8 Hz), alpha (8–13 Hz), beta (13–30 Hz), and gamma (>30 Hz). This filtering process also removes large artifacts like blinking and muscle movements. The filter is implemented using a finite impulse response (FIR) approach in MATLAB.

#### 2.2.2 Artifact subspace reconstruction process

Removing artifacts is a crucial step in the preprocessing of EEG data. For this task, we employed the artifact subspace reconstruction (ASR) technique, which automatically detects and utilizes clean segments of EEG recordings to eliminate noisy components. ASR is an automated, online, component‐based artifact removal method for removing transient or large‐amplitude artifacts in multi‐channel EEG recordings.^34^

One of the advantages of ASR over traditional filtering methods is its ability to preserve the integrity of the EEG signal more effectively. The ASR algorithm consists of five main stages. First, it identifies clean EEG segments by analyzing the distribution of signal variance to define a reference subspace free from noise. Second, it decomposes the signal into subspaces using principal component analysis (PCA). Third, based on the variance distribution within the principal component space, rejection criteria are then established. Fourth, the algorithm subsequently identifies components containing artifacts and either accepts or rejects them according to the defined thresholds. Finally, instead of discarding contaminated segments, ASR reconstructs the artifact‐laden components by projecting them back onto the clean reference subspace, thus retaining the underlying neural information. In this study, we employed the open‐source EEGLAB plugin clean_rawdata to apply ASR to the output of the initial filtering step, enabling artifact correction while maintaining the fidelity of the EEG signal.

To further refine the preprocessing pipeline and enhance signal quality, additional steps were applied following the ASR process. Although ASR effectively reduces transient and large‐amplitude artifacts, some residual noise, particularly from eye blinks, muscle activity, and cardiac signals, may still persist. To address this, independent component analysis (ICA) was performed as a complementary step, allowing the identification and removal of these remaining artifacts. The ICA algorithm was implemented using the EEGLAB toolbox in MATLAB, and independent components associated with artifacts were manually inspected and rejected. Additionally, to mitigate the impact of motion artifacts, amplitude thresholding was employed, where extreme voltage fluctuations deviating significantly from baseline activity were identified and removed. These additional preprocessing steps further improved the signal‐to‐noise ratio (SNR), ensuring that the retained EEG signals more accurately reflected neural activity relevant to emotion recognition.

### 2.3 Data segmentation

To extract data corresponding to the five emotional categories presented to the participants, 4.8334 min of data were selected from each video. Approximately 10 s of data following the end of each video were excluded, based on the assumption that participants may have experienced fatigue. The starting point for data extraction was aligned with the actual onset of each video, taking into account both audio and visual components. For the first video, which elicited the emotion of relaxation, data extraction began at the 5th second of playback. For the remaining four videos, data were extracted starting from the 1st second.

After segmentation the raw data, the dimension of each selected data matrix was (64*72,501). The rows of this matrix show the channel numbers and the columns of the recorded samples. This stage of data separation was done after the preprocessing operation for the recorded data of all 23 participants in the experiment and the output matrices were saved. In Equation (2‐1), E1, E2, E3, E4, and E5 show the 20th subject's selected data and S20 is 20th subject's raw data. After data segmentation, the recorded signal of each channel was separated from the output matrix of the previous step. According to the size of the matrix obtained in the previous step, the length of the matrix of each channel was 72,501. The output data of each channel was used as the main data in the next steps.

(2‐1) $$\left(\begin{array}{l} E1=S20\left(:,\;16250:88750\right);\text{Selected}\;\text{data}\;\text{for}\;\text{20th}\;\text{subject}’s\;\text{relaxation}\;\text{class}\\ E2=S20\left(:,\;106250:178750\right);\text{Selected}\;\text{data}\;\text{for}\;\text{20th}\;\text{subject}’s\;\text{happiness}\;\text{class}\\ E3=S20\left(:,\;196250:268750\right);\text{Selected}\;\text{data}\;\text{for}\;\text{20th}\;\text{subject}’s\;\text{motivation}\;\text{class}\\ E4=S20\left(:,\;286250:358750\right);\text{Selected}\;\text{data}\;\text{for}\;\text{20th}\;\text{subject}’s\;\text{sadness}\;\text{class}\\ E5=S20\left(:,\;376250:448750\right);\text{Selected}\;\text{data}\;\text{for}\;\text{20th}\;\text{subject}’s\;\text{fear}\;\text{class} \end{array}\right).$$

### 2.4 Feature extraction

**Figure 3:**
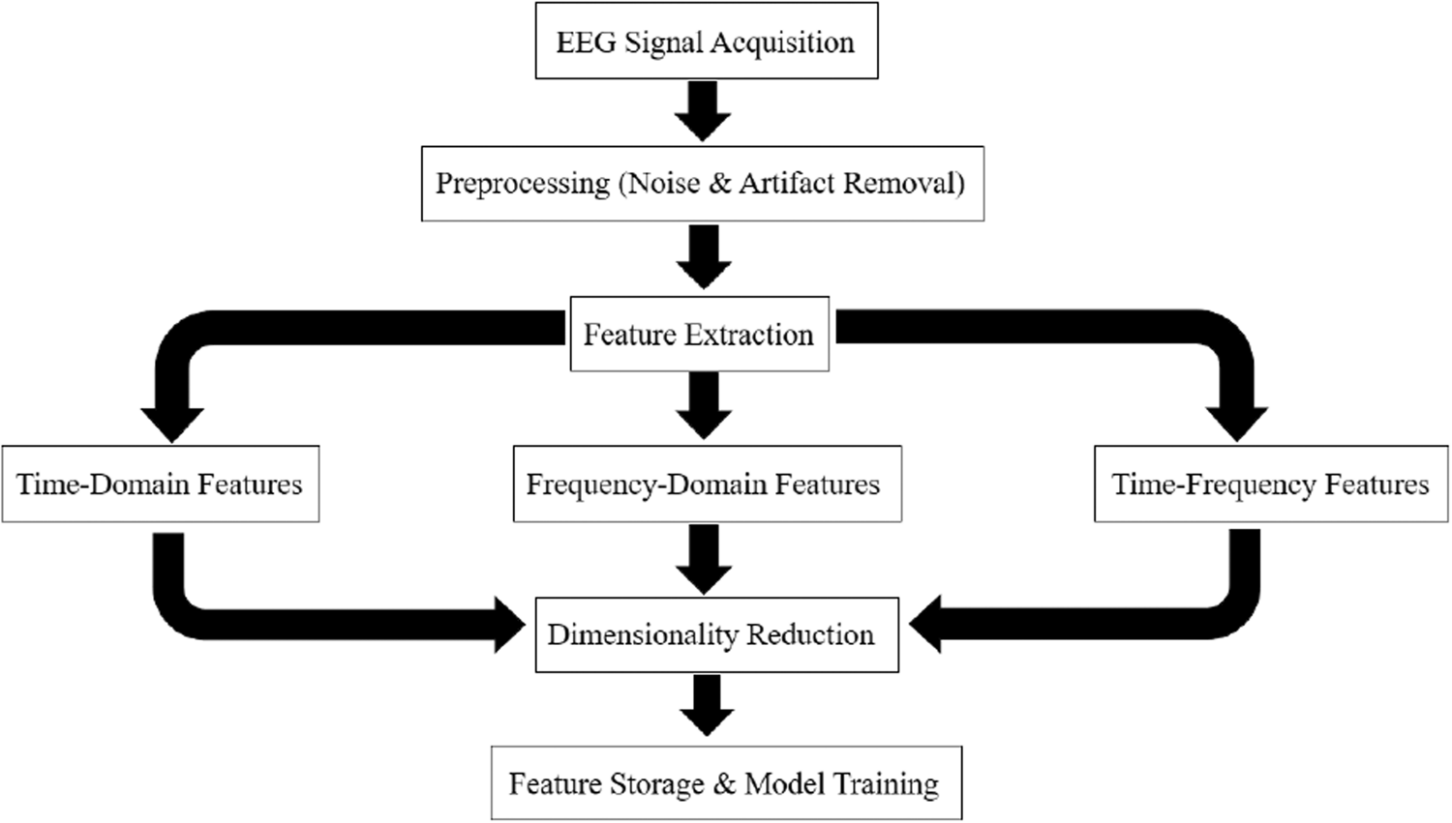
The processing pipeline of EEG signals. The steps include signal acquisition, preprocessing for noise and artifact removal, feature extraction from time‐domain, frequency‐domain, and time‐frequency representations, optional dimensionality reduction, and finally, feature storage and model training.

Feature extraction is a process in machine learning and data analysis that involves identifying and extracting relevant features from raw data. These features are later used to create a more informative data set, which can be further utilized for various tasks such as: classification, prediction and clustering. The feature is an individual measurable property within a recorded data set. Feature extraction aims to reduce data complexity, often known as “data dimensionality,” while retaining as much relevant information as possible. This helps to improve the performance and efficiency of machine learning algorithms and simplify the analysis process. Figure 3 illustrates the feature extraction process from EEG signals. It begins with EEG signal acquisition, followed by preprocessing to remove noise and artifacts. Features are extracted in three domains: time‐domain (statistical, time series, model‐based, rotating, nonlinear), frequency‐domain (spectral), and time‐frequency domain (spectrogram, empirical mode decomposition [EMD]). If necessary, dimensionality reduction is applied before storing the extracted features for machine learning model training.

After performing preprocessing in the feature extraction stage for each class, 54 statistical and frequency features were extracted from the data using linear and nonlinear algorithms. We extracted the feature matrix for each class. Considering that our sampling rate was 250 Hz in the signal recording stage and we selected 4.8334 min of data related to each class as the target data, so 72,501 samples were recorded by each channel. To have a suitable feature extraction, we considered the frame size and frame rate to be 50, and in every 50 samples, 54 statistical and frequency features were obtained. At the end, 1450 feature samples were extracted for each emotion class. The information of all the extracted features is provided separately in Supporting Information S1: Tables 3 and 4.

### 2.5 Feature selection

Feature selection plays a crucial role in this type of experiment, as it aids in identifying the most relevant features for enhancing model classification. For this purpose, we have chosen an inference‐based approach to feature selection, as selecting statistically significant features has been demonstrated to improve prediction accuracy in EEG data analysis,^35^ underscoring the importance of meticulous feature extraction for EEG‐based classification tasks. In this study, a statistical test is employed to assess whether significant differences exist between the five emotion classes for each feature. The following hypotheses are tested, where the symbols and represent the means of the data for the five emotion classes. H₀ denotes the null hypothesis, Ha represents the alternative hypothesis, and *μᵢ* refers to the mean of the data for each emotion class.

$$\begin{array}{l} H0:\mu 1=\mu 2=\mu 3=\mu 4=\mu 5\\ \;\text{Ha}:\;\text{At}\;\text{least}\;\;\mu i\;\text{different}. \end{array}$$

To conduct this test, we first examine whether the data from all five emotion classes follow a normal distribution and check for homoscedasticity, meaning whether the variances are equal. A significance level of *α* = 0.05 is set for these tests. Initially, normality is evaluated using Anderson‐Darling's test. If the data from all emotion classes conform to a normal distribution, Levene's test is subsequently applied to assess homoscedasticity, testing the null hypothesis that all samples have equal variances. After performing these tests, we select the features or columns where there is sufficient evidence to reject the null hypothesis, that is, the features exhibiting statistically significant differences between the class means (*p*‐value).

Once normality and homoscedasticity are established, a statistical test is applied to determine if the data means significantly differ. If all class data are normal with equal variances, we proceed with the analysis of variance (ANOVA) test. If the data are normal but variances are unequal, Welch's *t*‐test is used. In cases where at least one class does not follow a normal distribution and variances are nonhomogeneous, the Kruskal–Wallis test is applied.

By default, these statistical comparisons were implemented to select the best features, optimize model performance, and improve classification accuracy. Statistical comparisons, including confidence intervals and ANOVA, have been added to validate the performance differences among the models. These additions strengthen the analysis in the results section. These statistical comparisons were used as initial settings before classification to select the best features.

The Kruskal–Wallis test is employed in multi‐class classification tasks for four key reasons. First, it helps assess the impact of features on different classes by identifying whether a particular feature exhibits statistically significant differences across multiple groups; features lacking such variation are likely less informative for classification. Second, unlike parametric tests such as ANOVA, the Kruskal–Wallis test is nonparametric and does not assume normality, making it especially suitable for data types like EEG signals, which often deviate from a normal distribution. Third, it is well‐suited for multi‐class problems, as it can handle comparisons among more than two independent classes—an advantage over tests like the Mann–Whitney *U* test, which are limited to two groups. Lastly, the Kruskal–Wallis test offers efficiency and simplicity in feature selection by reducing the feature set without relying on more complex methods such as PCA or mutual information.

After applying Anderson, Darling, and Levin tests, we found that the data of all extracted features are not normal and homogeneous. This type of distribution is common in real data such as EEG signal. Because the data were not normal, a nonparametric test should be used. In the first step, using Kruskal–Wallis which is a nonparametric test, we selected the features with the appropriate significance level. In the second step, to ensure the results of this test, we repeated the feature selection step once again in the classification step, and by removing the features one by one and repeating the classification and evaluating the performance of the classifiers, we tried to identify the features that do not have a sufficient level of significance. In this way, we can be sure of the results of the Kruskal–Wallis test. After performing this step, we rejected six features that did not have a sufficient level of significance and selected a total of 48 features that had a sufficient level of significance for the emotion classification stage. The list of six rejected features is given in Table 2.

**Table 2:** Information of all rejected features.

No	Rejected featured
1	tsmodel/Freq. 2 (24th feature in Supporting Information S1: Table 3)
2	tsmodel/Damp2 (26th feature in Supporting Information S1: Table 3)
3	spectrogramfeat/PeakValue (41th feature in Supporting Information S1: Table 3)
4	spectrogramfeat/CrestFactor (42th feature in Supporting Information S1: Table 3)
5	spectrogramfeat/ImpulseFactor (43th feature in Supporting Information S1: Table 3)
6	spectrogramfeat/ClearanceFactor (44th feature in Supporting Information S1: Table 3)

**Figure 4:**
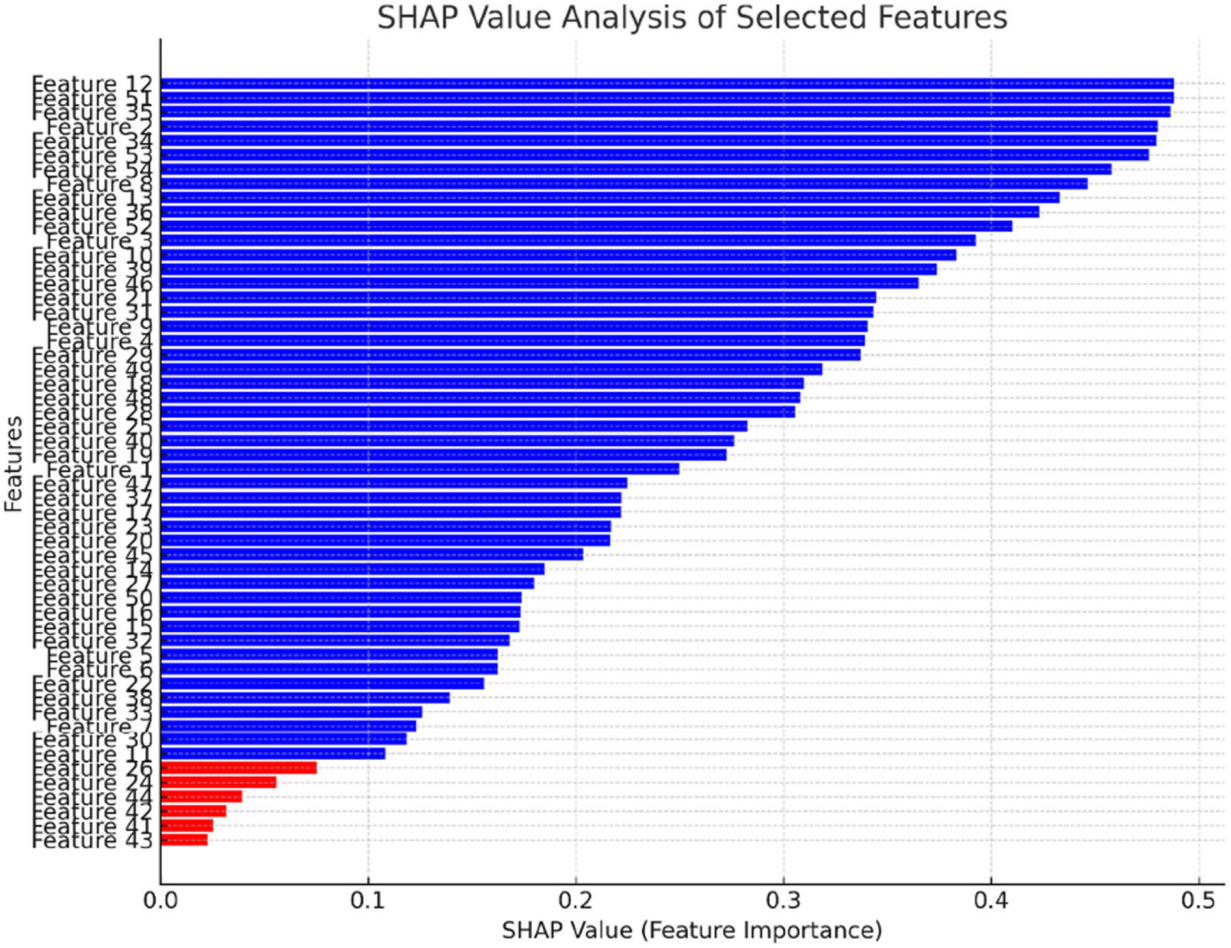
The shapley additive explanations (SHAP) value analysis of the selected features. Features marked in **red** (24, 26, 41, 42, 43, and 44) have SHAP values below 0.1, indicating low significance and were rejected. The remaining features (in **blue**) contribute significantly to the classification model. [Color figure can be viewed at wileyonlinelibrary.com]

What is more, the Shapley Additive Explanations (SHAP) value analysis was also conducted. The SHAP values indicate the importance of each feature in influencing the model's predictions. Features with higher SHAP values contribute more significantly to the classification, whereas those with lower values have a minimal impact. Figure 4 presents the SHAP value analysis for the selected features, highlighting their contribution to the classification process.

In this analysis, 54 features were evaluated, among which features 24, 26, 41, 42, 43, and 44 (marked in red) exhibited SHAP values below 0.1, indicating low significance. These features were rejected due to their insufficient contribution to the classification model. Conversely, the remaining features (marked in blue) demonstrated higher SHAP values, confirming their importance in distinguishing between emotional states. This analysis further validates the feature selection process, ensuring that only the most informative features are retained for classification, ultimately enhancing model performance and interpretability.

### 2.6 Data labeling

Because we use supervised learning algorithms, it is necessary to use data labeling. Supervised learning, a subset of machine learning and artificial intelligence, involves training algorithms using labeled data sets. These algorithms are designed to classify data or predict results with high accuracy. Considering that in this project we sought to identify and classify five classes, we should give a special label to the samples of the feature matrix of each emotion. As a result, the matrices of the selected features will be distinguished from each other and the classification models will have higher accuracy and better performance in the next stage. The selected features of different classes were organized as follows:

$$\begin{array}{l} \left[\text{selected}\;\text{features}\;\text{of}\;\text{first}\;\text{class}\right]1450*48\to e1(\text{first}\;\text{class}\;\text{label})\\ \left[\text{selected}\;\text{features}\;\text{of}\;\text{second}\;\text{class}]1450*48\to e2(\text{second}\;\text{class}\;\text{label}\right)\\ \left[\text{selected}\;\text{features}\;\text{of}\;\text{third}\;\text{class}]1450*48\to e3(\text{third}\;\text{class}\;\text{label}\right)\\ \left[\text{selected}\;\text{features}\;\text{of}\;\text{fourth}\;\text{class}]1450*48\to e4(\text{fourth}\;\text{class}\;\text{label}\right)\\ {}[\text{selected}\;\text{features}\;\text{of}\;\text{fifth}\;\text{class}]1450*48\to e5(\text{fifth}\;\text{class}\;\text{label}). \end{array}$$

### 2.7 Classification of EEG data

#### 2.7.1 Validation data

In the final stage, the matrix of the selected features that were labeled are used as input for classification models. To increase the reliability of project classification models, we used cross‐validation (5 folds). In modeling, particularly in machine learning, estimating model parameters is often required. When the number of parameters is high, the model's complexity grows, making calculations more challenging. Cross‐validation is a technique used to optimally select the number of model parameters (variables). It helps prevent overfitting by splitting the data set into several folds and assessing accuracy on each fold.

#### 2.7.2 Test data

After constructing the machine learning models using training data, it is essential to evaluate their performance on unseen data, referred to as the testing set. This evaluation helps assess the generalization capability of the models and guides further optimization. In our study, we experimented with different training/testing splits and observed the performance of the classifiers under identical conditions. The best classification performance was achieved when 80% of the data set was used for training and the remaining 20% was allocated for testing. Therefore, in the final implementation, we adopted this 80/20 split to ensure both reliability and consistency in model evaluation.

#### 2.7.3 Classification models

**Figure 5:**
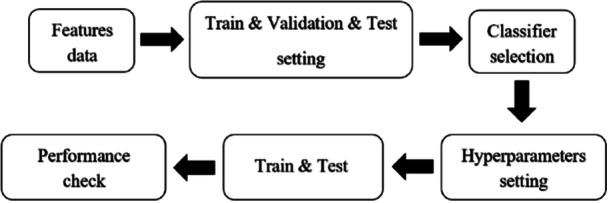
All components of the classification data.

In the next step, we used eight classification models for training and testing the data. The eight classification models are: decision tree, linear discriminant analysis (LDA), Naive Bayes, support vector machine (SVM), K‐nearest neighbor (KNN), ensemble, logistic regression, and neural network. We optimized the classification model by changing the parameters and kernels of each model with 100 iterations. After classifying the five emotion classes, we compared the performance accuracy of the models. We did this separately for all 64 channels of the 23 participants in the project. Figure 5 shows the different parts of classification steps.

## 3 RESULTS

### 3.1 Classification results

Due to the fact that the EEG signal was recorded with 64 channels, we perform the emotion classification steps channel by channel separately for all 23 people participating in the experiment. The main reason is that the channels have correlation and we cannot analyze the data of all channels together. In the following, as an example, the results of the emotion classification operation for the 15th channel (C3) of the 20th subject are given. After classification for all channels of a subject, the final accuracy of each subject was obtained with the average accuracy of all channels.

**Figure 6:**
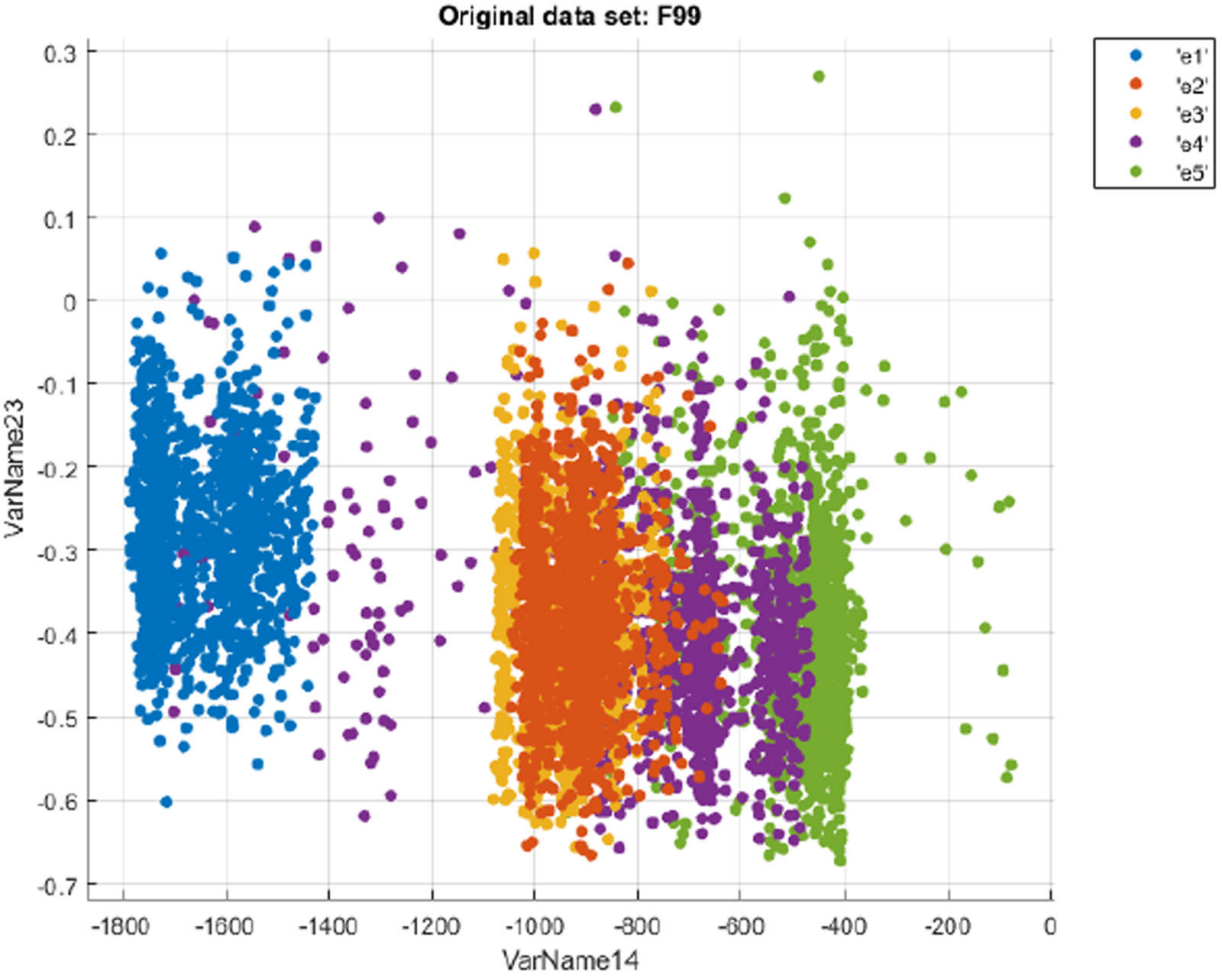
Input data for the 15th channel (C3) of the 20th subject. VarName14 represents the 14th feature (tsfeat/Minimum), and VarName23 represents the 23rd feature (tsmodel/Freq. 1). [Color figure can be viewed at wileyonlinelibrary.com]

For the 15th channel (C3) of the 20th subject the scatter plot of the data is given in Figure 6. Features 14 and 23 were randomly selected from the pool of extracted features for the purpose of schematic illustration and visualization. These features were not chosen based on statistical or classification performance criteria, but rather to provide clear graphical representation and separation in visual plots. Therefore, their selection does not reflect any underlying preference or importance in the classification results.

### 3.2 Decision tree

The decision tree is a widely used machine learning algorithm for both classification and regression tasks. It is a model that resembles a flowchart, helping to predict outcomes by associating observations of an item with conclusions about its target variable. A decision tree consists of nodes that represent data attributes, branches that indicate decision rules, and leaves that signify outcomes or class labels. Essentially, the decision tree algorithm builds a tree‐like structure from the training data, illustrating the series of decisions that lead to predicting the target variable. The algorithm works by repeatedly splitting the data into smaller subsets based on features that provide the most information, continuing this process until a final decision or outcome is reached.^36^

**Figure 7:**
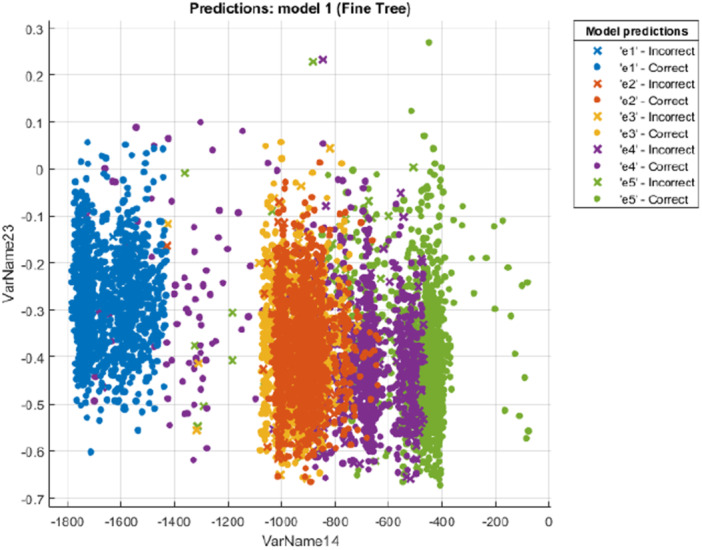
Decision tree model prediction for the 15th channel (C3) of the 20th subject. VarName14 represents the 14th feature (tsfeat/Minimum), and VarName23 represents the 23rd feature (tsmodel/Freq. 1). [Color figure can be viewed at wileyonlinelibrary.com]

The first algorithm we used to classify the five classes of emotions in this project was the “decision tree.” For the 15th channel (C3) of the 20th subject by adjusting the hyperparameters and after 100 iterations the accuracy of this model reached 91.7%. Figure 7 shows the scatter plot of the data separately for each class in different colors which the horizontal axis is the (tsfeat/Minimum) and the vertical axis is (tsmodel/Freq. 1). In each class, the samples that were correctly recognized by the model are in the form of circular dots, and the samples that were not recognized correctly by the model are shown with a sign of multiplication.

To improve the performance and increase the accuracy of the decision tree model, we use three types of decision tree and tried to optimize the model with 100 iterations. The three types of used decision tree are fine tree, medium tree and coarse tree. The fine tree has many leaves and can make many distinctions between classes (the maximum number of splits is 100). A medium tree is a flexible decision tree with fewer leaves (the maximum number of splits is 20) and a coarse tree is a simple decision tree with very few leaves that make coarse distinctions between classes (the maximum number of splits is 4). After optimizing and adjusting the hyperparameters and repeating the classification, we realized that the fine tree has the best performance.

### 3.3 LDA

LDA is a statistical technique used to reduce the dimensionality of a problem while identifying groups by maximizing the ratio of “between‐group scatter” to “within‐group scatter.” The approach of linear discriminant analysis is closely related to the method developed by Ronald Fisher, which was originally used to assess differences between groups and later formed the foundation for analysis of variance.^37^ The classification output of this model is presented in Supporting Information S1: Figure 1, where an accuracy of 84.9% was achieved.

We use two types of LDA and tried to optimize the model with 100 iterations. The two types of used LDA are linear discriminant and quadratic discriminant. The linear discriminant is a fast and easy discriminant classifier to interpret, which creates linear boundaries between classes. The quadratic discriminant is a fast and easy discriminant classifier to interpret, which creates elliptical, parabolic or hyperbolic boundaries between classes. After optimizing and adjusting the hyperparameters and repeating the classification, we realized that the linear discriminant has the best performance.

### 3.4 Naive Bayes

Naive Bayes is a widely used classification algorithm in machine learning. It is a probabilistic model that relies on Bayes' theorem to estimate the likelihood of an event occurring, based on prior knowledge of conditions related to the event. The key assumption of Naive Bayes is that the features of the input data are independent of one another, which simplifies probability calculations and enhances computational efficiency. The algorithm learns the conditional probability of each feature for every class from the training data. When given new, unseen data, Naive Bayes calculates the probability for each class based on the observed features and predicts the class with the highest probability. Despite its simplicity and the assumption of feature independence, Naive Bayes performs well in various classification tasks, particularly when the training data is limited relative to the number of features.^38^ The classification results of this model are shown in Supporting Information S1: Figure 2, with an accuracy of 80.8%.

We use two types of this model and tried to optimize the model with 30 iterations. The two types of used Naive Bayes are Gaussian Naive Bayes and kernel Naive Bayes. The Gaussian Naive Bayes uses Gaussian distribution for numeric predictors and multivariate multinomial (MVMN) distribution for categorical predictors, while the kernel Naive Bayes uses kernel distribution for numeric predictors and MVMN distribution for categorical predictors. After optimizing and adjusting the hyperparameters and repeating the classification, we realized that the Gaussian Naive Bayes has the best performance.

### 3.5 SVM

SVM is a supervised learning technique that uses labeled training data to find the optimal hyperplane for classifying new data into distinct categories. SVM is valuable because it can manage high‐dimensional datasets, performs well with small sample sizes, and is effective across various applications. Additionally, SVM is capable of handling nonlinear data by utilizing kernel functions, which allow it to capture complex patterns within the data.^39^ The model's performance, visualized in Supporting Information S1: Figure 3, indicates an achieved accuracy of 88.1%.

We use six types of this model and tried to optimize the model with 30 iterations, including linear, quadratic, cubic, fine Gaussian, medium Gaussian and coarse Gaussian. The linear SVM makes a simple linear separation between classes, using the linear kernel. It's the easiest SVM to interpret. The quadratic SVM uses the quadratic kernel and cubic SVM uses the cubic kernel. The fine Gaussian SVM makes finely detailed distinctions between classes, using the Gaussian kernel with kernel scale set to sqrt(P)/4, where P is the number of predictors (features). Medium Gaussian SVM makes fewer distinctions than a fine Gaussian SVM, using the Gaussian kernel with kernel scale set to sqrt(P), where P is the number of predictors (features). The coarse Gaussian SVM makes coarse distinctions between classes, using the Gaussian kernel with kernel scale set to sqrt(P)*4, where P is the number of predictors (features). After optimizing and adjusting the hyperparameters and repeating the classification, we realized that the medium Gaussian SVM has the best performance.

### 3.6 KNN

The KNN algorithm is a straightforward supervised machine learning technique commonly used for classification and regression tasks. In classification, it identifies the *k* nearest neighbors and predicts the class based on the majority vote of those neighbors. The algorithm evaluates the similarity between new data points and existing ones, then assigns the new data to the class that is most similar. KNN is considered nonparametric, meaning it makes no assumptions about the data distribution. Additionally, it is referred to as a “lazy learning” algorithm because it doesn't learn from the training data right away but instead stores the data and processes it during classification.^40^ This model achieved a classification accuracy of 79.6%, as shown in Supporting Information S1: Figure 4.

We use six types of KNN and tried to optimize the model with 30 iterations, including fine, medium, coarse, cosine, cubic and weighted KNN. The fine KNN makes finely detailed distinctions between classes, with the number of neighbors set to one. The medium KNN makes fewer distinctions than a fine KNN, with the number of neighbors set to 10. The course KNN makes coarse distinctions between classes, with the number of neighbors set to 100. The cosine KNN uses the cosine distance metric, while cubic KNN uses the cubic distance metric and weighted KNN uses distance weighting. After optimizing and adjusting the hyperparameters and repeating the classification, we realized that the fine KNN has the best performance.

### 3.7 Ensemble classifier

**Figure 8:**
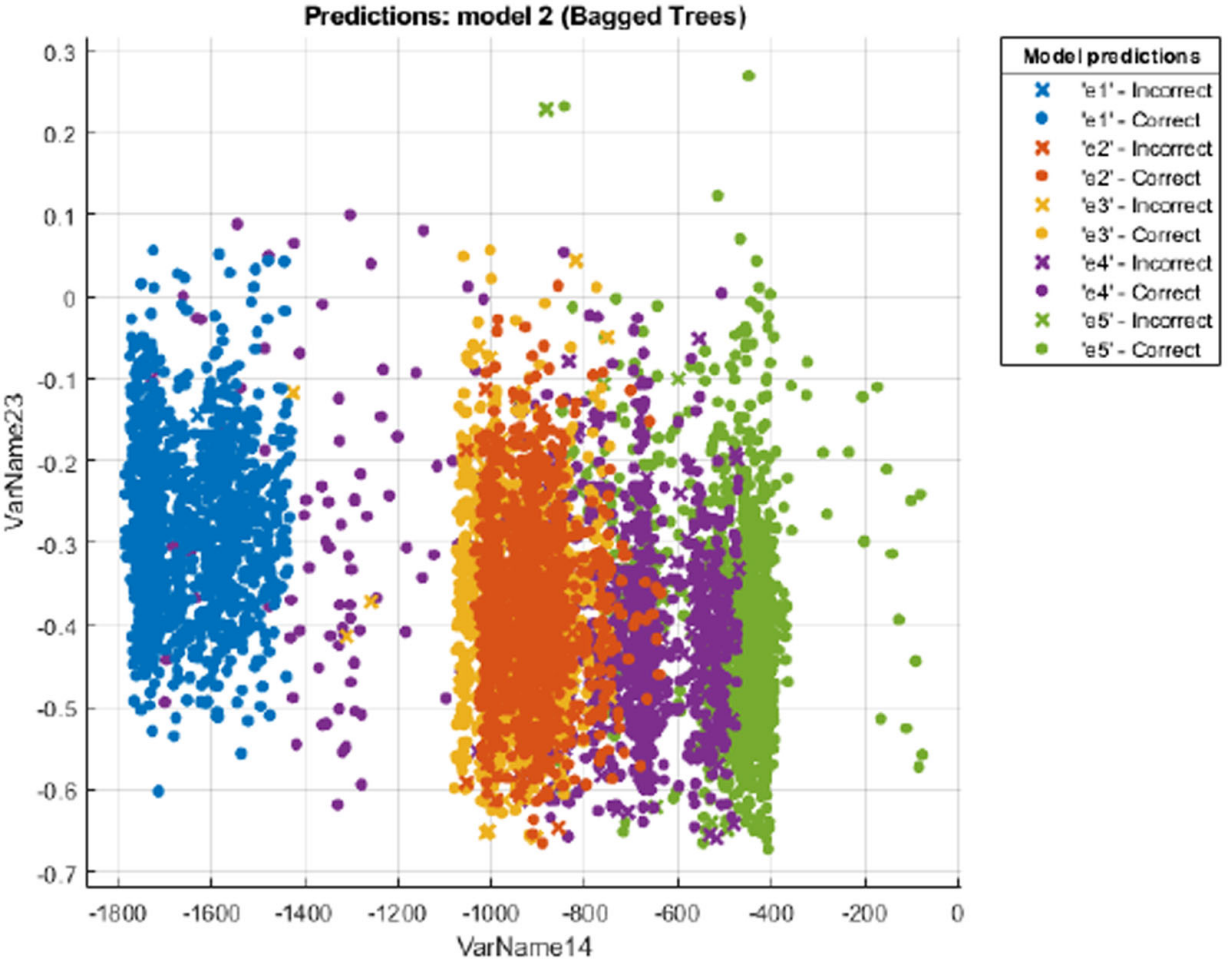
Ensemble model prediction for the 15th channel (C3) of the 20th subject. VarName14 represents the 14th feature (tsfeat/Minimum), and VarName23 represents the 23rd feature (tsmodel/Freq. 1). [Color figure can be viewed at wileyonlinelibrary.com]

Ensemble models are a machine learning technique where multiple models, often referred to as weak learners or base models, are trained to address a problem, with the goal of combining their results for improved performance. When these weak models are effectively integrated, they can generate more accurate or consistent predictions. In ensemble methods, these basic models serve as the foundation for building more sophisticated models. Typically, these individual models may not perform well independently due to high bias or variance.^41^ The classification results obtained by this model are presented in Figure 8, with an accuracy of 94.1%.

We utilized five different ensemble techniques and performed 30 iterations for model optimization. The five types of used ensemble included boosted trees, bagged trees, subspace discriminant, subspace KNN and RusBoosted trees. The boosted tree creates an ensemble of medium decision trees using AdaBoost algorithm. The bagged tree is a bootstrap aggregated ensemble of fine decision trees. It's often accurate, but can be slow and memory intensive for large datasets. Compared to bagging, boosting algorithms use relatively little time or memory, but might need more ensemble members. The subspace discriminant is good for many predictors, relatively fast for fitting and prediction, and low on memory usage, but the accuracy varies depending on the data. This model is an ensemble of discriminant classifiers using the random subspace algorithm. Similarly, the subspace KNN model, which constructs an ensemble of nearest neighbor classifiers using the same algorithm, is particularly suitable for high‐dimensional datasets. The RusBoosted trees model is specifically designed to handle imbalanced datasets, where one class significantly outweighs the others in sample size. It combines boosting with random undersampling to improve performance under such conditions. After hyperparameter tuning and repeated classification trials, the bagged trees model was found to deliver the best overall performance among the five ensemble techniques evaluated.

### 3.8 Logistic regression

Logistic regression is a type of supervised machine learning algorithm primarily used for classification tasks. Its main objective is to estimate the probability that a data point belongs to a specific class. This algorithm, which has a statistical foundation, explores the connection between independent variables and a binary dependent variable. It is widely applied in decision‐making scenarios, such as determining whether an email is spam or not. Although known for classification, the algorithm is named “logistic regression” because it incorporates the output of a linear regression model as input but uses the sigmoid function to calculate the probability of belonging to a particular class. The key distinction between linear and logistic regression is that linear regression generates a continuous output, whereas logistic regression predicts the likelihood of a sample belonging to one of the two possible classes.^42^ The classification outcome is shown in Supporting Information S1: Figure 5, with an accuracy of 71.7%.

We used an efficient logistic regression model and tried to optimize the model with 100 iterations. Efficient logistic regression is a linear logistic regression classifier that uses techniques that reduce the model training time when the training data set includes many predictors and many observations.

### 3.9 Neural network

Neural networks, often referred to as artificial or simulated neural networks, are key elements of machine learning and the foundations of deep learning models. These networks are designed to replicate the human brain's processing abilities. Structured similarly to the brain, they consist of interconnected units known as neurons, which perform data processing and analysis tasks. At the core of each neural network is the neuron, which takes in input signals, processes them, and generates output. Neural networks are structured in layers, with each layer playing a unique role in the learning process.

Neurons serve as the fundamental units within a neural network, taking in inputs, performing calculations, and sending out results. In an artificial neural network, neurons are modeled using mathematical functions that compute a weighted average of the inputs. These functions, through an activation process, determine the output of each neuron. The network fine‐tunes the weights of the neuron connections via the backpropagation algorithm to minimize the difference between the predicted and actual outputs. By repeating this process across numerous examples, the network gradually learns to make precise predictions.^43^ The results of this model, showing an accuracy of 87.7%, are illustrated in Supporting Information S1: Figure 6.

We use five types of neural network and tried to optimize the model with 100 iterations. The five types are narrow, medium, wide, bilayered and trilayered neural network. The narrow neural network is a classifier with one fully connected layer of size 10. The medium neural network is a classifier with one fully connected layer of size 25. The wide neural network is a classifier with one fully connected layer of size 100. The bilayered neural network is a classifier with two fully connected layers, excluding the final fully connected layer for classification. The trilayered neural network is a classifier with three fully connected layers, excluding the final fully connected layer for classification. After optimizing and adjusting the hyperparameters and repeating the classification, we realized that the narrow neural network model has the best performance.

### 3.10 Comparison of models

The performance of the classification models was evaluated using fivefold cross‐validation. The table below shows the performance accuracy of eight models used to identify five different human emotions. The average final accuracy of the models was calculated according to Equations (3‐1) and (3‐2) where *M* is the average of each channel, *A* is the average accuracy of the model per person participating in the project, and *T* is the final accuracy of the model. In Equation (3‐1), 64 are the number of channels and in Equation (3‐2), 23 are the number of people participating in the project. After calculations, the ensemble and decision tree models had the highest accuracy with 95.38% and 91.77%. The results of all classification models are given in Table 3.

(3‐1) $$A=\frac{\sum_{c=1}^{64}\mathrm{Mc}}{64},$$

(3‐2) $$T=\frac{\sum_{s=1}^{23}\mathrm{As}}{23}.$$

**Table 3:** Results of all classification models.

Classification model	Average validation accuracy (%)	Average test accuracy (%)
Decision tree	90.49	91.77
LDA	85.43	84.90
Naive Bayes	80.49	80.92
SVM	88.52	88.33
KNN	80.23	79.86
Ensemble	94.12	95.38
Logistic regression	71.68	71.82
Neural network	86.51	87.71

Abbreviations: KNN, K‐nearest neighbor; LDA, linear discriminant analysis; SVM, support vector machine.

## 4 DISCUSSION

Emotion recognition using EEG signals plays a vital role in enhancing human–computer interaction and deepening our understanding of affective states that influence cognition and behavior.^44^ In this study, we developed a comprehensive emotion classification framework by evaluating eight machine learning models on EEG data collected from 23 participants exposed to emotionally evocative video stimuli.

Our results demonstrate that ensemble and decision tree classifiers achieved the highest accuracies of 95.38% and 91.77%, respectively, outperforming other classical classifiers such as logistic regression and Naive Bayes. The superior performance of ensemble methods can be attributed to their ability to integrate multiple base learners, thereby improving the generalization and robustness of the classification system against the inherent variability and noise present in EEG signals. Likewise, decision trees provide interpretability and effectively model nonlinear feature interactions, which are essential for capturing the complex neural dynamics associated with emotional states.

When compared to previous research (Table 4), our classification accuracies are competitive, especially considering the larger number of emotion categories (five classes) and the wide range of machine learning algorithms assessed. Although some deep learning approaches report slightly higher accuracies, our findings emphasize the continued effectiveness of classical machine learning techniques when combined with rigorous feature extraction and selection procedures.

**Table 4:** Comparison of our study with previous works.

Study	Classification models	Classification accuracy (%)
Our study	Decision Tree, LDA, Naive Bayes, SVM, KNN, Ensemble, Logistic Regression, Neural Network	71.82–95.38
Mouazen et al. (2025)^45^	KNN, SVM, Decision Tree, RF, BiLSTM, GRU, CNN, Transformer	Classical machine learning models: 56.2–60.9
		Recurrent neural networks models: 91–94
Chen et al. (2024)^46^	Multi‐Scale Dynamic 1D CNN, Gated Transformer	DEAP: 99.66
		SEED: 98.85
Li et al. (2025)^47^	Spatial and temporal Transformers	SEED: 92.67
Liu et al. (2024)^48^	Hybrid CNN, Transformer	DEAP: 74.23
		SEED: 67.17
Du et al. (2024)^49^	Capsule Transformer	DEAP: 98.31
		SEED: 94.91
Zhang et al. (2024)^50^	TPRO‐NET	DEAP: 97.47–97.88
		DREAMER: 98.18–98.40

Abbreviations: BiLSTM, bidirectional long short‐term memory; CNN, convolutional neural network; DEAP, database for emotion analysis using physiological signals; DREAMER, EEG and ECG data set for emotion recognition; GRU, gated recurrent unit; KNN, K‐nearest neighbor; LDA, linear discriminant analysis; RF, random forest; SEED, SJTU emotion EEG data set; SVM, support vector machine.

One might question whether a sample size of 23 participants is sufficient to support the claim that brain signals change in response to emotions induced by videos. While a larger sample size could improve generalizability, numerous previous studies on EEG‐based emotion recognition have reported significant results with similar or even smaller sample sizes.^51^, ^52^ Additionally, EEG experiments often involve complex data collection and analysis, making studies with smaller but well‐controlled samples a common approach in neuroscience research.^53^ Furthermore, it is well established that human emotions are closely linked to physiological signals, particularly brain and heart activity. Studies have shown that external stimuli can meaningfully alter neural oscillations, leading to measurable changes in EEG signals associated with emotional states.^54^, ^55^ Therefore, while our study is based on 23 participants, the observed patterns are consistent with well‐documented findings in the field, suggesting that the results contribute meaningfully to the broader understanding of emotion‐induced neural responses.

Nevertheless, this study faces certain limitations. While the sample size of 23 participants aligns with many EEG‐based emotion recognition studies and is sufficient to identify significant patterns, it may still constrain the broader applicability of the results to more heterogeneous populations. EEG data collection is resource‐intensive, and variability between subjects remains a significant challenge. Future work should investigate domain adaptation strategies and personalized modeling to enhance robustness across diverse populations.

Furthermore, physiological and neural responses to emotions are complex and influenced by individual differences, making EEG signal interpretation challenging. Our study contributes to this understanding by demonstrating that advanced classification algorithms can successfully decode multiple emotional states from EEG features, highlighting the potential for practical applications in affective computing and brain–computer interface technologies.

In conclusion, this study provides a thorough comparison of multiple classification models for EEG‐based emotion recognition, revealing that ensemble and decision tree methods offer superior accuracy and interpretability. These insights lay the foundation for the development of more reliable and adaptive affective technologies, ultimately facilitating enhanced human‐machine interactions. symbiosis. Table 4 presents the methods and results of studies similar to ours. In terms of the diversity of classification models used, data set size, and final classification accuracy, our study outperforms many comparable works.

## 5 CONCLUSION

In this study, EEG signals were used to classify five emotional states: relaxation, happiness, motivation, sadness, and fear. Data from 23 participants were analyzed using statistical and frequency‐domain features, and eight different machine learning classifiers were evaluated. The ensemble model achieved the highest performance with an average test accuracy of 95.38%, followed by the decision tree (91.77%) and SVM (88.33%). In contrast, logistic regression showed the lowest performance at 71.82%. These results demonstrate the effectiveness of ensemble‐based and tree‐based models for EEG‐based emotion recognition.

## AUTHOR CONTRIBUTIONS

Afshin S. Asl contributed to the conceptualization and design of the study, developed the methodology, implemented the software, collected the data, and drafted the original manuscript. Sahar Karimpour contributed to the conceptualization and methodology of the study and provided critical revisions to the manuscript.

## CONFLICT OF INTEREST STATEMENT

The authors declare no conflicts of interest.

## ETHICS STATEMENT

This study was conducted in accordance with the Declaration of Helsinki. Ethical approval was obtained from the Ethics Committee of Tabriz University (Approval Code: IR.TABRIZU.REC.1398.030). Written informed consent was obtained from all participants before their inclusion in the study. Participants were informed that their anonymized data may be used in research publications.

## Supporting information

Supplementary_Materials.

## Data Availability

The data sets generated and/or analyzed during the current study are available from the corresponding author on reasonable request.
