# Supplementary material for: Emotional recognition while watching emotional videos: Based on electroencephalography signal analysis and machine learning models
Source: Ibrain. 2025 Sep 19;11(3):347–63. doi: 10.1002/ibra.70002 (PMC12465226; doi:10.1002/ibra.70002)
Supplement: Supplementary file 1 — Supplementary_Materials. [file IBRA-11-347-s001.docx]

**Supplementary materials**

This supplementary document includes additional figures and tables that support the main results of the paper. These materials provide extra details on the experimental procedures, model outputs, and data analyses, which complement the findings discussed in the main text.

**Supplementary figures**


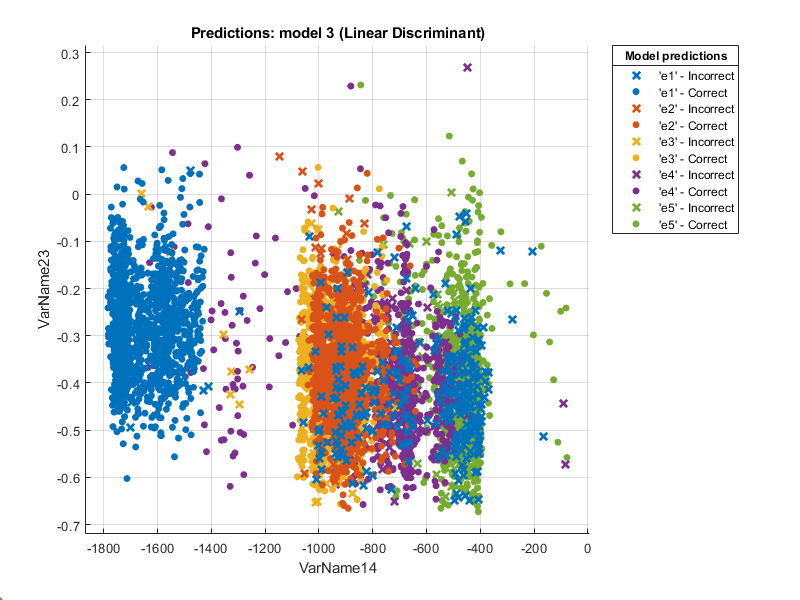


Figure 1 LDA model prediction for the 15th channel (C3) of the 20th subject. VarName14 represents the 14th feature (tsfeat/Minimum), and VarName23 represents the 23rd feature (tsmodel/Freq1).


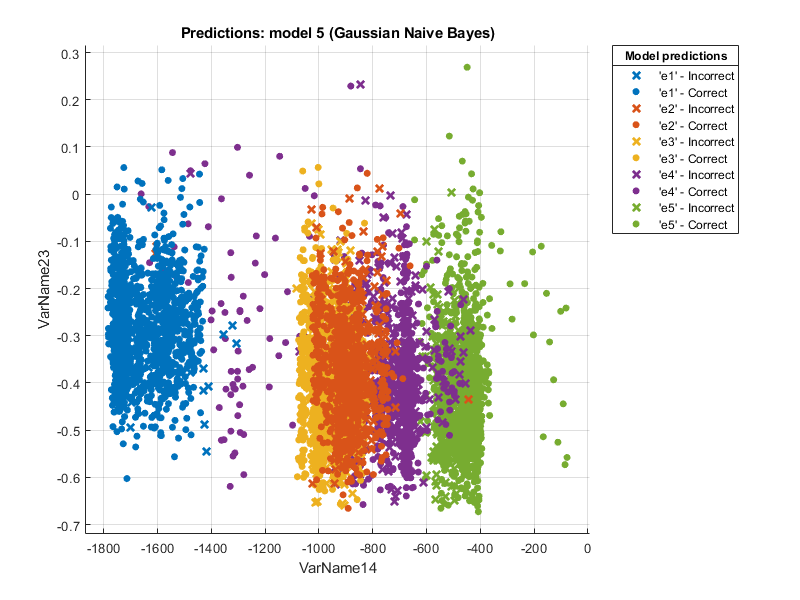


Figure 2 Naive Bayes model prediction for the 15th channel (C3) of the 20th subject. VarName14 represents the 14th feature (tsfeat/Minimum), and VarName23 represents the 23rd feature (tsmodel/Freq1).


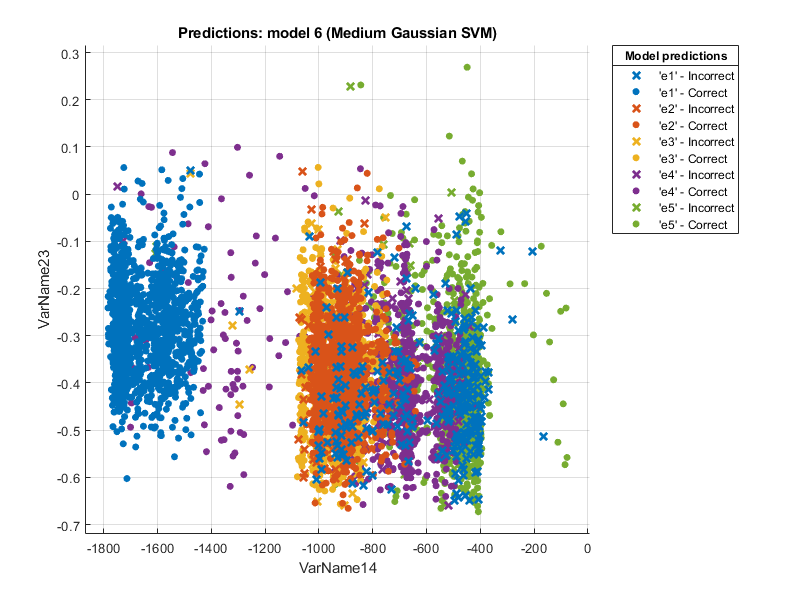


Figure 3 SVM model prediction for the 15th channel (C3) of the 20th subject. VarName14 represents the 14th feature (tsfeat/Minimum), and VarName23 represents the 23rd feature (tsmodel/Freq1).


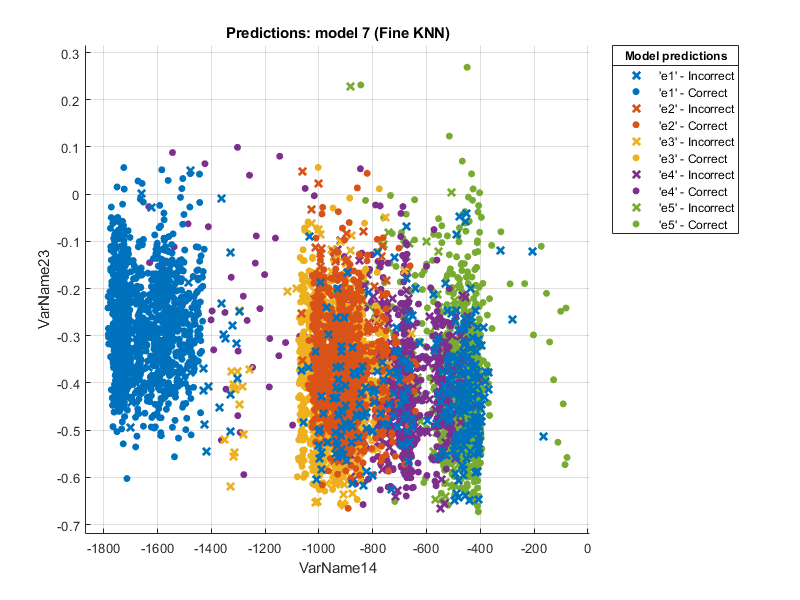


Figure 4 KNN model prediction for the 15th channel (C3) of the 20th subject. VarName14 represents the 14th feature (tsfeat/Minimum), and VarName23 represents the 23rd feature (tsmodel/Freq1).


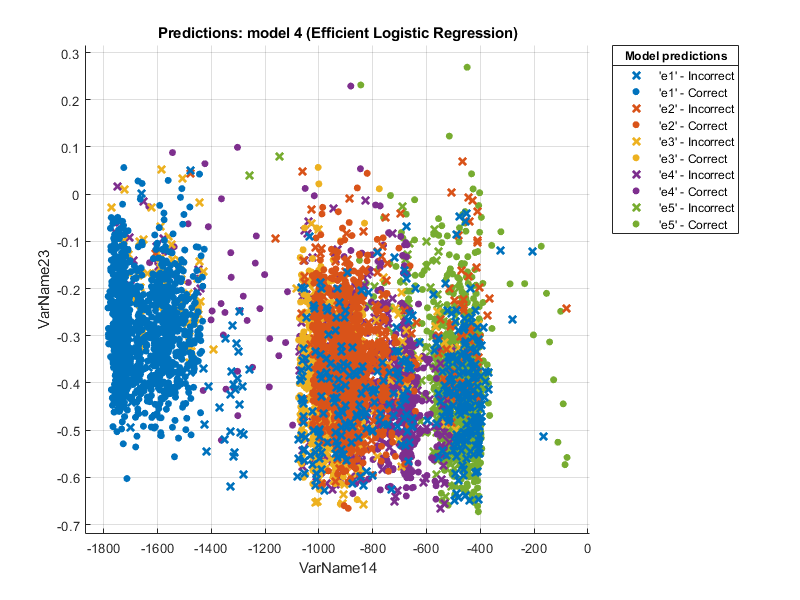


Figure 5 Logistic regression model prediction for the 15th channel (C3) of the 20th subject. VarName14 represents the 14th feature (tsfeat/Minimum), and VarName23 represents the 23rd feature (tsmodel/Freq1).


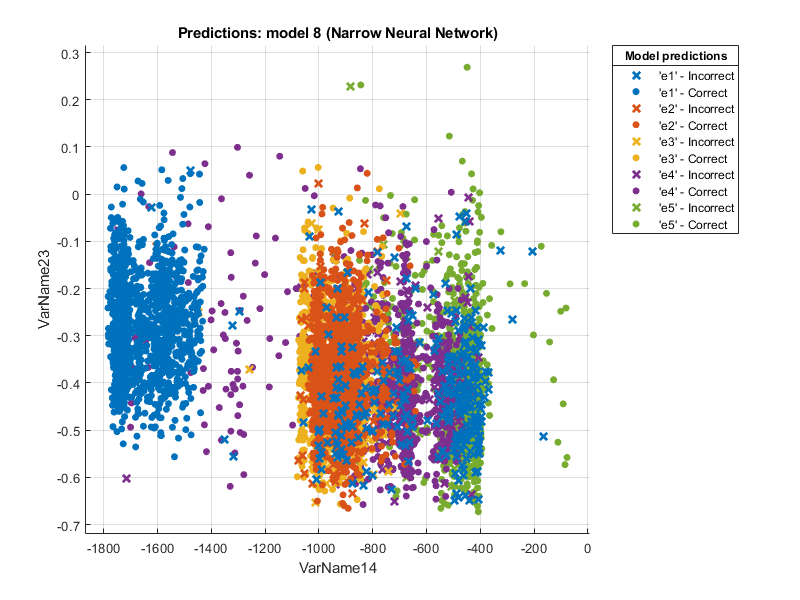


Figure 6 Neural network model prediction for the 15th channel (C3) of the 20th subject. VarName14 represents the 14th feature (tsfeat/Minimum), and VarName23 represents the 23rd feature (tsmodel/Freq1).

**Supplementary tables**

Table 1 Description of experimental protocol.

| Number of subjects | Subject’s age range | Number of channels | Sampling frequency (Hz) | Low frequency (Hz) | High frequency (Hz) |
| --- | --- | --- | --- | --- | --- |
| 23 | 24-31 | 64 | 250 | 0.3 | 50 |

Table 2 The used channels to recording EEG signal.

| **Channel**  **number** | **Label** | **Channel**  **number** | **Label** | **Channel**  **number** | **Label** | **Channel**  **number** | **Label** |
| --- | --- | --- | --- | --- | --- | --- | --- |
| **1** | 'FP1' | **17** | 'C4' | **33** | 'AF7' | **49** | 'CPz' |
| **2** | 'FPz' | **18** | 'T8' | **34** | 'AF3' | **50** | 'CP4' |
| **3** | 'FP2' | **19** | 'A2' | **35** | 'AF4' | **51** | 'P5' |
| **4** | 'F7' | **20** | 'CP5' | **36** | 'AF8' | **52** | 'P1' |
| **5** | 'F3' | **21** | 'CP1' | **37** | 'F5' | **53** | 'P2' |
| **6** | 'Fz' | **22** | 'CP2' | **38** | 'F1' | **54** | 'P6' |
| **7** | 'F4' | **23** | 'CP6' | **39** | 'F2' | **55** | 'PO5' |
| **8** | 'F8' | **24** | 'P7' | **40** | 'F6' | **56** | 'PO3' |
| **9** | 'FC5' | **25** | 'P3' | **41** | 'FC3' | **57** | 'PO4' |
| **10** | 'FC1' | **26** | 'Pz' | **42** | 'FCz' | **58** | 'PO6' |
| **11** | 'FC2' | **27** | 'P4' | **43** | 'FC4' | **59** | 'FT7' |
| **12** | 'FC6' | **28** | 'P8' | **44** | 'C5' | **60** | 'FT8' |
| **13** | 'A1' | **29** | 'POz' | **45** | 'C1' | **61** | 'TP7' |
| **14** | 'T7' | **30** | 'O1' | **46** | 'C2' | **62** | 'TP8' |
| **15** | 'C3' | **31** | 'Oz' | **47** | 'C6' | **63** | 'PO7' |
| **16** | 'Cz' | **32** | 'O2' | **48** | 'CP3' | **64** | 'PO8' |

Table 3 All extracted features.

| **No** | **Feature name** | **No** | **Feature name** | **No** | **Feature name** |
| --- | --- | --- | --- | --- | --- |
| **1** | sigstats/ClearanceFactor | **19** | tsmodel/Coef2 | **37** | nonlin/ApproxEntropy |
| **2** | sigstats/CrestFactor | **20** | tsmodel/Coef3 | **38** | nonlin/CorrelationDim |
| **3** | sigstats/ImpulseFactor | **21** | tsmodel/Coef4 | **39** | nonlin/LyapunovExp |
| **4** | sigstats/Kurtosis | **22** | tsmodel/Coef5 | **40** | spectrogramfeat/SpectralEntropy |
| **5** | sigstats/Mean | **23** | tsmodel/Freq1 | **41** | spectrogramfeat/PeakValue |
| **6** | sigstats/PeakValue | **24** | tsmodel/Freq2 | **42** | spectrogramfeat/CrestFactor |
| **7** | sigstats/RMS | **25** | tsmodel/Damp1 | **43** | spectrogramfeat/ImpulseFactor |
| **8** | sigstats/SINAD | **26** | tsmodel/Damp2 | **44** | spectrogramfeat/ClearanceFactor |
| **9** | sigstats/SNR | **27** | tsmodel/MSE | **45** | emdfeat/EnergyIMF1 |
| **10** | sigstats/ShapeFactor | **28** | tsmodel/MAE | **46** | emdfeat/PeakValueIMF1 |
| **11** | sigstats/Skewness | **29** | tsmodel/AIC | **47** | emdfeat/CrestFactorIMF1 |
| **12** | sigstats/Std | **30** | tsmodel/Mean | **48** | emdfeat/ImpulseFactorIMF1 |
| **13** | sigstats/THD | **31** | tsmodel/Variance | **49** | emdfeat/ClearanceFactorIMF1 |
| **14** | tsfeat/Minimum | **32** | tsmodel/RMS | **50** | ps_spec/PeakAmp1 |
| **15** | tsfeat/Median | **33** | tsmodel/Kurtosis | **51** | ps_spec/PeakFreq1 |
| **16** | tsfeat/Maximum | **34** | rotmac/RMS | **52** | ps_spec/Wn1 |
| **17** | tsfeat/ACF1 | **35** | rotmac/Kurtosis | **53** | ps_spec/Zeta1 |
| **18** | tsmodel/Coef1 | **36** | rotmac/CrestFactor | **54** | ps_spec/BandPower |

Table 4 Information of all extracted features.

| All Extracted Features | | |
| --- | --- | --- |
| **Time-Domain features** | **Time-Frequency-Domain features** | **Frequency-Domain features** |
| Signal statistical (1-13) | Spectrogram (40-44) | Spectral (50-54) |
| Time series (14-17) | EMD (45-49) |  |
| Model-based (18-33) |  |  |
| Rotating machinery (34-36) |  |  |
| Nonlinear (37-39) |  |  |
